# Oral Bone Tissue Regeneration: Mesenchymal Stem Cells, Secretome, and Biomaterials

**DOI:** 10.3390/ijms22105236

**Published:** 2021-05-15

**Authors:** Agnese Gugliandolo, Luigia Fonticoli, Oriana Trubiani, Thangavelu S. Rajan, Guya D. Marconi, Placido Bramanti, Emanuela Mazzon, Jacopo Pizzicannella, Francesca Diomede

**Affiliations:** 1 Department of Experimental Neurology, IRCCS Centro Neurolesi “Bonino-Pulejo”, Via Provinciale Palermo, Contrada Casazza, 98124 Messina, Italy; agnese.gugliandolo@irccsme.it (A.G.); placido.bramanti@irccsme.it (P.B.); 2 Department of Innovative Technologies in Medicine & Dentistry, University “G. d’Annunzio” Chieti-Pescara, Via dei Vestini, 31, 66100 Chieti, Italy; luigia.fonticoli@unich.it (L.F.); oriana.trubiani@unich.it (O.T.); francesca.diomede@unich.it (F.D.); 3 Department of Biotechnology, School of Life Sciences, Karpagam Academy of Higher Education, Coimbatore 641021, India; tsrajanpillai@gmail.com; 4 Department of Medical, Oral and Biotechnological Sciences, University “G. d’Annunzio” Chieti-Pescara, Via dei Vestini, 31, 66100 Chieti, Italy; guya.marconi@unich.it; 5 “Ss. Annunziata” Hospital, ASL 02 Lanciano-Vasto-Chieti, 66100 Chieti, Italy; jacopo.pizzicannella@unich.it

**Keywords:** tissue engineering, regenerative medicine, biomaterials, biocompatibility, scaffold, tissue repair, dentistry

## Abstract

In the last few decades, tissue engineering has become one of the most studied medical fields. Even if bone shows self-remodeling properties, in some cases, due to injuries or anomalies, bone regeneration can be required. In particular, oral bone regeneration is needed in the dentistry field, where the functional restoration of tissues near the tooth represents a limit for many dental implants. In this context, the application of biomaterials and mesenchymal stem cells (MSCs) appears promising for bone regeneration. This review focused on in vivo studies that evaluated bone regeneration using biomaterials with MSCs. Different biocompatible biomaterials were enriched with MSCs from different sources. These constructs showed an enhanced bone regenerative power in in vivo models. However, we discussed also a future perspective in tissue engineering using the MSC secretome, namely the conditioned medium and extracellular vesicles. This new approach has already shown promising results for bone tissue regeneration in experimental models.

## 1. Introduction

In healthy conditions, bone tissue shows a self-remodeling property due to resorption and new bone formation processes that are modulated by osteoclast and osteoblast activities. However, several bone injuries or anomalies are difficult to heal by natural body processes, causing the necessity for bone regeneration strategies. Bone regeneration is also sometimes required in the dentistry field when dental implants are needed. Indeed, tooth loss is one of the major problems related to a decrease of life quality. The success of dental implants also depends on the functional restoration of tissues near the tooth, including bone. For this reason, the regeneration of the bone is of fundamental importance in dentistry [1,2].

In this context, the main purpose of regenerative medicine is to overcome the limit of the low regenerative power of tissues, creating strategies to restore their functionality and architecture. Tissue engineering denotes a novel approach to regenerative medicine in order to develop tissue-like complexes that mimic the role and structural organization of tissues in order to repair injuries, anatomical defects, and restore the functions of the damaged areas using biomaterials and stem cells [3,4,5]. Specifically, the differentiation capacity and the paracrine effects of stem cells, such as mesenchymal stem cells (MSCs), are useful for tissue regeneration, and their application seems promising in different fields of regenerative medicine, including bone regeneration [6,7,8]. Moreover, their combination with scaffolds may have positive effects on the regeneration of many damaged tissues. Scaffolds are three-dimensional structures that promote the reconstruction of tissue architecture. The scaffold is made of a biomaterial that provides a template to ameliorate the healing process and to promote cell attachment, proliferation, differentiation, and extracellular matrix (ECM) generation [5,9]. Obviously, scaffolds need to show physical features that allow for oxygen and nutrient transport, the maintenance of cell survival, and the promotion of cell differentiation and homing in order to allow for MSC growth. Interestingly, in recent years, cell-free strategies have gained substantial attention in the world of tissue engineering [10]. In this context, scaffolds can be combined with MSC products, namely their secretome.

In this review, we focused on in vivo studies evaluating the potential application of MSCs combined with biomaterials for bone regeneration, with a particular interest in biomaterials and MSC constructs that can be applied in the dentistry field. We also evaluated the cell-free approach based on the enrichment of biomaterials with the MSC secretome as a promising future perspective.

## 2. Mesenchymal Stem Cells and Regenerative Medicine

MSCs are undifferentiated cells known for their self-renewal and differentiation properties. MSCs are also able to secrete immunomodulatory factors, leading to the creation of a regenerative microenvironment. Thanks to these properties, MSCs may play a main role in regenerative medicine, and some clinical studies have demonstrated that MSCs from different sources may have the ability to repair injured tissues [7].

The Mesenchymal and Tissue Stem Cell Committee of the International Society for Cellular Therapy stated the minimal criteria to define MSCs: MSCs must be plastic-adherent; express CD105, CD73, and CD90; lack the expression of CD45, CD34, CD14, CD11b, CD79alpha, or CD19 and HLA-DR surface molecules; and are able to differentiate toward osteoblasts, adipocytes, and chondroblasts in vitro [11]. MSCs also have the ability to trans-differentiate into cells of the different germ layers: mesoderm lineage cells, as well as ectoderm and endoderm lineage cells [6].

Being multipotent adult stem cells, MSCs show less moral, ethical, or safety problems compared to embryonic stem cells [6]. Furthermore, these cells possess an immunomodulatory action and poor immunogenicity. Bone marrow-derived MSCs (BMSCs) were the first to be discovered, but MSCs can be isolated from multiple sources, including adipose and oral tissues [12]. Oral mesenchymal stem cells (OMSCs) can be easily isolated from the tissues residing in the oral cavity with less invasive techniques compared to BMSCs, which require bone marrow collection [13,14,15]. In particular, from dental tissues, dental pulp stem cells (DPSCs), stem cells from the apical papilla (SCAPs), periodontal ligament stem cells (PDLSCs), gingival-derived MSCs (GMSCs), dental follicle stem cells (DFSCs), tooth germ stem cells (TGSCs), and alveolar bone-derived MSCs (ABMSCs) were isolated [14].

The paracrine effects of MSCs have attracted a lot of attention in tissue regeneration. Specifically, MSCs can secrete different growth and trophic factors, cytokines, and microRNAs, known as the secretome that includes both the conditioned medium (CM) and extracellular vesicles (EVs) [10,16,17]. Different proteins, such as vascular endothelial growth factor (VEGF), transforming growth factor β (TGF-β), and bone morphogenetic proteins (BMPs), all directly involved in bone regeneration, have been already found to be released by MSCs in the CM or inside EVs [10,18]. However, both proteins and non-coding RNA contained in EVs were found to participate in bone regeneration [19]. Both CM and EVs may be used for cell-free therapy, and their applications seem promising in clinical practice, not showing the ethical limits related to the use of stem cells [20].

In tissue reparative treatments, cells or their secretome can be directly applied to the damaged area or be associated to scaffolds that promote cell attachment, proliferation, differentiation, ECM generation, and the diffusion of biomolecules inducing the restoration of tissues.

## 3. Biomaterials and Scaffold Characteristics for Bone Tissue Engineering

In the last few decades, the definition of biomaterials was the main topic of various scientific debates. In 1987, the European Society for Biomaterials (ESB) defined biomaterials as non-viable materials used in medical devices or implants that are able to interact with biological systems, such as tissues or organs, but their definition has changed over the years [21].

Of main importance is that a biomaterial must be biocompatible, allowing for cell attachment without the stimulation of an immune response. Specifically, for bone tissue engineering, a biomaterial needs to have good tensile and compressive strength and to be osteoconductive and osteoinductive. Specifically, an osteoconductive biomaterial stimulates bone cell growth, while an osteoinductive one induces the proliferation and differentiation of MSCs [5]. A good biomaterial should also be resorbed when new bone is formed so that it can replace it. Moreover, the degradation byproducts should not be toxic.

Given the close relationship between osteogenic and angiogenic events [22], a good construct for bone regeneration should also improve angiogenesis [23]. Indeed, new blood vessel formation, promoted by growth factors, is necessary for minerals and oxygen transport, supports cell growth and interaction, and takes part in osteointegration processes [24].

Various biomaterials have been identified and used for bone regeneration. The most frequently used are ceramics, synthetics, and natural polymers and composites.

Ceramics are inorganic, non-metallic substances. Ceramics are classified as non-inert or resorbable (calcium phosphates and calcium aluminates), semi-inert (glass-ceramics and dense hydroxyapatites), and relatively inert (alumina, zirconia, silicone nitrides, and carbons). In dentistry, the use of ceramics is related to their high toughness and biocompatibility, although they are vulnerable to stress tensile and can be damaged due to excessive mechanical stress [25]. Calcium phosphates are among the most used biomaterials, given that the bone inorganic matrix is formed by calcium phosphates, even if they show a low tensile strength. Hydroxyapatite (HA) use is widespread due to its similarity to the inorganic matrix of the bone [5]. It presents a long resorption time but a reduced mechanical resistance. Β-tricalcium-phosphate (β-TCP) can be more easily produced than HA and also shows a faster resorption time, but it is more fragile [9].

Polymers can be divided into natural polymers, including collagen (Col), alginate, and chitosan, and synthetic polymers such as polylactic acid (PLA) and polycaprolactone (PCL) [26]. Natural polymers can be divided in protein-based ones, such as Col, and polysaccharide-based ones, including alginate and chitosan. Among natural polymers, the most used is Col, a considerable structural element of the bone matrix that is widely used in regenerative medicine [27]. To date, 28 different types of Col have been identified, but types I, II, and III are the most common and represent the classical fibril-forming Col [28]. Type I Col is the main organic constituent of the bone ECM and is widely used in bone tissue engineering [29]. Col shows a low antigenic response, a high tensile strength, and a high flexibility, but its degradation rate can cause a loss of mechanical strength. Gelatin (Gel) is obtained by the partial hydrolysis of Col and comparatively shows a lower antigenicity. Chitosan is a polysaccharide present in crustacean and insect exoskeletons, as well as in mushrooms. It shows an osteoconductive capacity but has the disadvantages of mechanical weakness and instability [5].

Synthetic polymers can be produced in controlled conditions, allowing for the production of scaffolds with specific physical and mechanical properties. PLA has a good tensile strength. Moreover, its hydrolytic degradation creates lactic acid. It is available in many forms such as poly(L-lactic acid) (PLLA) and poly(d,l-lactic acid) (PDLA). PCL is characterized by biocompatibility and slow degradation. Polylactic-co-glycolic acid (PLGA) is a copolymer of PLA and polyglycolic acid (PGA). PLGA is biodegradable, allows for cell adhesion, and shows good mechanical properties. Its degradation rate can be regulated by varying the percentage of the two polymers [30].

The combination of biomaterials (composites) is often used in the regenerative treatments. In this way, each biomaterial can bring features lacking in other ones, and the advantageous properties can be combined in order to obtain specific chemical, physical, and mechanical properties [31]. Composites are made of a polymer phase, with toughness and compressive strength, and an inorganic one that improves the mechanical properties and degradation rate [32]. Specifically, composites may show improved mechanical stability, better flexibility, and structural integrity. The result is that composite biomaterials may have a combination of the best properties of each biomaterial and other useful properties not shown by their constituents alone [33].

Scaffolds are three-dimensional structures made of biomaterials designed to promote cell–biomaterial interactions, cell adhesion, survival, proliferation, differentiation, and ECM deposition, as well as to allow for the transport of gases and nutrients and to provoke a minimal degree of inflammation or toxicity in vivo [34]. Some scaffolds, other than providing a mechanical support for cells, are considered “bioactive,” acting as vehicles for various molecules, such as cytokines, growth factors, drugs, and enzyme inhibitors that promote bone regeneration [35,36]. Depending on the application, scaffolds need to be designed appropriately. Mechanical, physical, and chemical properties allow scaffolds to be as similar as possible to tissues, while their architectures must provide structural support. These properties depend on the biomaterial and design of the scaffold. However, choosing the best scaffold components and selecting the best protocols for their development are still controversial topics.

In porous scaffolds, the density and size of pores influence cell migration and adherence to the scaffolds, as well as nutrient and oxygen diffusion. The pore dimension has been extensively investigated to induce bone growth, but there is not a clear consensus about the optimal pore size. Small pores may avoid cell penetration to the center of the scaffold, while in large pore scaffolds, the area available for cell attachment may be limited and cells may not be able to bridge the gaps between the pores. Moreover, an excessive porosity can make a scaffold too weak [37,38]. It was reported that a small pore size (<100 µm) is associated with the formation of non-mineralized osteoid or fibrous tissue, while pores over 100 μm are generally preferred for cell infiltration and bone growth [39]. However, it is important to notice that the time factor is important for cell adhesion. Indeed, an experimental study showed that the number of adherent cells significantly increased with time. At earlier time points, cells were found on the surface of the scaffold, while at later time points, cells were able to penetrate into the inner part of the scaffold [40].

Fibrous scaffolds mimic the fibrous nature of Col. Fiber may vary in size and organization. Hydrogels represent hydrophilic polymer networks formed through the crosslinking of monomer or polymer chains through covalent and/or noncovalent interactions. They show physical and chemical properties similar to some human tissues [41,42]. By modifying the crosslinking method and degree, it is possible to obtain the desired geometry, degradation rate, porosity or release profile [43].

The identification of the correct biomaterials for scaffold construction is important in order to create the best response to host tissue and to avoid the activation of an inflammatory response [44].

Moreover, surface modifications of scaffolds may modulate the scaffolds’ features, thus influencing their interactions with MSCs. Changes in surface topography, functional groups, and wettability were investigated [38], and various mechanical and chemical techniques have been proposed in order to modify the number of functional groups, surface charge, hydrophilicity, and molecular weight of compounds [45].

## 4. Biomaterials and Mesenchymal Stem Cells for Bone Tissue Engineering

In dentistry, dental implants are used to replace missing or damaged teeth in order to re-establish the correct relationship in the cranio-facial complex, but the lack of adequate bone support may impair implant functionality. For this reason, tissue engineering in dentistry has received a lot of interest [46]. Numerous studies have been carried out using MSCs and biomaterials for bone regeneration. We performed a PubMed search looking for studies using in vivo experimental models published from 2016 involving the use of MSCs, biomaterials, bone regeneration, and dentistry. Rats, mice, rabbits, and dogs are the most used animal models where alveolar bone or mandibular defects were induced. However, in addition to oral bone defect models, extraoral bone defect models have been created to evaluate bone regeneration with application in the dentistry fields. Specifically, the extraoral sites can offer an easily available amount of bone volume and an easier surgical approach. In this context, tibial or femoral defect models were used [47]. Additionally, the mouse calvaria model may be helpful for a variety of disease applications in the field of dental research and may provide data about bone regeneration [48].

### 4.1. In Vivo Studies Using MSCs and Scaffolds for Bone Regeneration

Studies were carried out in order to evaluate the efficacy of scaffolds enriched with MSCs for bone regeneration. A summary of the studies described in this section is present in Table 1.

A chitosan/anorganic bovine bone (C/ABB) scaffold seeded with human jaw bone marrow-derived MSCs (JBMMSCs) showed greater new bone and cementum formation compared to a control. The evaluation of osteocalcin by immunohistochemistry indicated the presence of osteogenesis in the C/ABB group with cells, as well as in the group transplanted with a chitosan scaffold and cells [49].

New bone formation was also visible in rats with critical-sized calvarial defects implanted with polydopamine (PDA)-laced HA Col calcium silicate (HCCS) with osteogenic MSC aggregates, while the scaffold without MSCs showed limited osteoconductivity. Additionally, dopamine plays a main role in bone regeneration given that MSC-seeded HCCS, without PDA, were found to induce less bone formation compared to the scaffold enriched with PDA [50].

In another study, Bio-Oss^®^ was tested in combination with concentrated growth factors (CGFs) or BMSCs on bone regeneration for maxillary sinus floor augmentation in beagle dogs. The new formed bone and hardness were similar in the two groups, while they were lower in the group with Bio-Oss^®^ alone, indicating that the newly formed bone in groups treated with Bio-Oss^®^ and BMSCs or CGFs may be more mature [51].

In another study, Laponite^®^ was crosslinked with *N*-isopropylacrylamide (NIPAM) and *N*,*N*′–dimethylacrylamide (DMAc) to form a hydrogel delivery system (L-pNIPAM-co-DMAc) loaded with HA nanoparticles (HAna). L-pNIPAM-co-DMAc implanted in a rat femur defect model for four weeks was shown to be biocompatible. In young male rats, bone defect repair seemed to be less effective when L-pNIPAM-co-DMAc seeded with MSCs was injected. In aged rats, histological analysis did not show a better repair when MSCs were incorporated within L-pNIPAM-co-DMAc and HAna. However an increase in runx2 was found in rats treated with L-pNIPAM-co-DMAC, HAna, and MSC, suggesting an improved osteogenicity when MSCs were injected [52].

A biomimetic tissue-engineered periosteum (TEP) composed of PCL, type I Col, and nano-HA composite nanofiber sheets seeded with BMSCs, combined with a bone allograft, restored donor-site periosteal bone formation, reversing the poor biomechanics of bone allograft healing [53].

Tonsil-derived MSCs mixed with Matrigel may represent also a valid approach for osteoradionecrosis, especially when applied immediately after damage. Indeed, bone regeneration and mineral density were found to be better in the group transplanted immediately after trauma with tonsil-derived MSCs compared to those receiving tonsil-derived MSCs after four weeks [54].

The regenerative capacity of a biphasic scaffold consisting of bone and periodontal ligament enriched with gingival cells, BMSCs, and periodontal ligament cells (PDLCs) was studied, and it was demonstrated to be well-integrated with the surrounding tissue in a periodontal defect model in sheep. The PDLC group showed enhanced bone formation compared with the empty scaffold, while the group with gingival cells showed less bone formation [55].

Another study, using co-culture of endothelial cells and MSCs, suggested that the distance between the two type of cells in 3D printed co-culture may influence angiogenesis and bone regeneration. When the cells in the scaffolds were separated by a distance of <200 μm, an elevated number of blood vessels was shown and major bone regeneration [56].

HA is among the most used biomaterials for scaffold construction. Interestingly, an HA scaffold enriched with stem cells from human exfoliated deciduous teeth (SHED) was proven to be an effective agent in alveolar bone defect regeneration, increasing osteoprotegerin (OPG) and decreasing RANKL, leading to a reduction of osteoclastogenesis [57]. Additionally, the association of an HA matrix with PLGA enriched with human DPSCs induced new bone formation and angiogenesis, leading to lesion size reduction in rabbits with bilateral, mandibular, critical-sized defects. Interestingly, the scaffold without DPSCs was less efficacious [58]. Another composite TCP–PLGA scaffold with osteogenic differentiated adipose-derived MSCs (ADMSCs) increased bone volume and osteocalcin deposition when integrated in the mandibular critical-sized defects in a minipig model. On the contrary, the scaffold without cells was less efficacious [59].

In another study, alginate was modified with dopamine and methacrylate residues, to increase its adhesive properties, and combined with HA microparticles. Specifically, to induce osteogenesis, HA microparticles were incorporated into GMSC aggregates, and the resulting HA/GMSC aggregates were encapsulated into the hydrogel. The hydrogel, subcutaneously implanted in mice, was shown to be biodegradable, biocompatible, and osteoconductive. Moreover, in a rat peri-implantitis model, the transplantation of hydrogel-encapsulating GMSCs induced complete bone regeneration [60].

Recently, an injectable, self-crosslinking, thiolated hyaluronic acid/type I Col I blend of hydrogel and biphasic calcium phosphate ceramic combined with BMSCs and chondrocytes was used to fabricate a new bi-layer scaffold. The BMSC/chondrocyte-loaded bi-layer scaffold promoted bone regeneration in an osteochondral defect model [61].

These studies indicated the efficacy of MSC-seeded scaffolds for bone regeneration in different in vivo models. Various biomaterials and MSC sources were used in these studies. Interestingly, some studies clearly showed that MSC-seeded scaffolds increase bone formation compared to unseeded ones, evidencing the success of the combination of scaffolds with MSCs.

### 4.2. In Vivo Studies Comparing Different Biomaterials or Scaffold Features in Association with MSCs for Bone Regeneration

With the aim to define the most appropriate biomaterials, some studies compared scaffolds made of different biomaterials, different composites, and different scaffold features. A summary of the studies described in this section is available in Table 2. Specifically, one study investigated the effects of three different scaffolds made of polyamide, PLGA, or decellularized amniotic membrane seeded with ADMSCs for bone regeneration of calvarial defects in a rabbit model. In the control group, the defect was covered with non-seeded scaffolds. Only minimal bone regeneration was visible after two weeks of wound healing, while it became more evident after four weeks in all groups compared to controls. A more complete defect closure was found in all the cell-seeded groups after eight weeks. However, the animals treated with polyamide scaffolds showed the best results. These results may indicate that MSC-seeded polyamide scaffolds may be more useful for bone regeneration [62]. Moreover, DPSCs with three different scaffolds, made of L-lactide and DL-lactide (PLDL), a copolymer of DL-lactide (PDL) or HA/TCP, respectively, were transplanted in mice. The expression of genes involved in different processes during odontogenic differentiation, such as dentin sialo-phosphoprotein (DSPP), dentin matrix protein-1 (DMP1), enamelysin/matrix metalloproteinase 20 (MMP20), and phosphate-regulating gene with homologies to endopeptidases on X chromosome (PHEX), were evaluated. A decreased expression of enamelysin/MMP20 was found in PLDL and HA/TCP at 12 weeks, while all other expressions increased and reached the greatest level at 12 weeks. The PDL group showed the greatest DSPP expression, while the HA/TCP group showed the greatest DMP1 expression. The greatest expression of PHEX was found in the PLDL group. Consequently, PLDL and PDL enriched with DPSC seem to be promising scaffolds for odontogenic regeneration, as does HA-TCP [63].

Another study evaluated the guided bone regeneration of a calvarial, critical-sized defect using BMSCs and a Col membrane with and without TCP material. The results showed that the hardness of the new bone was similar to the native bone in the groups with the Col membrane with and without cells. Instead, the bone in the group treated with Col membrane, TCP, and BMSCs showed a higher elasticity [64].

In composite scaffolds, the different percentages of biomaterials may influence bone regeneration. ABMSCs were transplanted using an HA/TCP scaffold or the slowly resorbing biomaterial Bio-Oss in a murine ectopic transplantation model. Specifically, different HA/TCP percentages were used: 20% HA/80% TCP and 60% HA/40% TCP. ABMSCs with HA/TCP scaffolds increased bone regeneration in the ectopic transplantation model with the significant increase of osteoblasts. On the contrary Bio-Oss did not induce new bone formation when loaded with ABMSCs. Positive immunostaining for the bone tissue formation markers ALP, RUNX-2, OCN, and OPN was observed with HA/TCP, and in particular, the 60% HA/40% TCP composite showed more positively stained cells. On the contrary, only a limited expression of osteogenic markers was evident with Bio-Oss. Conversely, there was a minimal osteoclast presence with Bio-Oss but a significant presence of osteoclasts with both 20% HA/80% TCP and 60% HA/40% TCP scaffolds [65].

However, in addition to biomaterials, scaffold features can influence bone formation. Interestingly, alginate hydrogels containing BMP-2 with smaller pores and greater elasticity may induce greater bone regeneration, preventing MSC apoptosis induced by pro-inflammatory cytokines. Indeed, SHED encapsulated in hydrogel with a greater elasticity showed a reduced expression of NF-kB p65 and Cox-2 in vivo. The results may indicate that the mechanical features and microarchitecture of the scaffold influenced the fate of the encapsulated MSCs [66].

It was shown that nanofibrous PLLA scaffolds seeded with BMSCs induced bone regeneration, and bone volume increased with the increase of pore size [67]. Similarly, nanocomposite PDA-laced HCCS scaffolds seeded with MSCs implanted in rats with critical-sized defect induced different bone regeneration levels depending on the pore dimension. New bone formation and bone regeneration were observed after the implantation of scaffolds with 500-μm-sized pores, while scaffolds with 250-μm-sized pores only induced minimal bone formation [68].

βTCP with nano-diamond particles induced more bone formation compared to βTCP alone because a nano-diamond coating is used to bind proteins to material surfaces and to enhance MSC attachment. The enrichment with MSCs resulted in little difference compared with the scaffold without cells [69].

A strontium-substituted, HA-based bioceramic scaffold (SrHAB) was tested for its capacity to induce bone regeneration. The scaffolds containing 10% of strontium induced bone formation and the osteogenic differentiation of MSC from human and ovine sources in ectopic bone formation in mice and in ovine models, respectively [70].

These studies evidenced that different biomaterials may be more useful for bone regeneration with MSCs. Interestingly, scaffold features may also influence the regenerative processes, even in the presence of MSCs. As such, the understanding of the best scaffold characteristics is important to create the best scaffold that will be used in clinical practice along with MSCs.

### 4.3. In Vivo Studies Using Scaffolds Enriched with Biomolecules and MSCs for Bone Regeneration

The enrichment of scaffolds with biomolecules, such as BMPs, can also improve bone regeneration. A summary of the studies described in this section is available in Table 3.

Khojasteh et al. evaluated the ability of MSCs delivered using TCP scaffolds in combination with endothelial progenitor cells (EPCs) and/or coated with PLGA microspheres releasing VEGF in the repair of dog mandible bone defects. Bone formation was greatest in the VEGF/MSC scaffold, followed by the VEGF/MSC/EPC and MSC/EPC scaffolds. The results indicated that new bone regeneration was greater in scaffolds containing MSCs in the presence of both EPC and VEGF. Moreover, osteoblast-like cells were often observed in the MSC-seeded groups. However, the greatest bone formation that occurred when the VEGF/MSC scaffold was implanted might have been due to the greater number of MSCs seeded in the VEGF-containing scaffolds than in those containing EPC [71].

Alginate-chitosan beads enriched with MSCs and BMP-2 or basement membrane proteins induced bone repair in a murine cranial non-union defect, narrowing the defect and inducing the formation of osteoid tissue [72].

An osteoinductive scaffold composed of BMP-6-loaded nano-HA (nHA)/Gel/Gel microsphere pre-seeded with MSCs was tested in critical-sized calvarial bone defects in rats. These scaffolds were cytocompatible and enhanced new bone formation. Interestingly, the new bone at the lesion site was larger with the BMP-6-loaded scaffolds compared with the BMP-6-free scaffolds [73]. A nHA/Col I/multi-walled carbon nanotube (MWCNT) composite scaffold loaded with recombinant BMP-9 enhanced bone formation in vivo. Specifically, the scaffold was able to stimulate new bone formation, and the enrichment with BMP-9 increased this effect [74]. Fahimipour et al. developed a construct with heparin-conjugated Col hydrogel immobilizing BMP-2, reinforced by 3D printed β-TCP-based bioceramic scaffold. In vivo mineralized tissue was found in rats implanted either with the scaffold enriched with MSCs and heparin or the scaffold with MSCs but without heparin. However, greater new bone formation was found when heparin was present. Moreover, BMP-2′s presence increased the expression of genes involved in osteogenesis [75]. Additionally, BMP-2-functionalized PCL biomembranes enriched with BMSCs implanted in a maxillary bone lesion were found to induce bone regeneration. In particular, the presence of BMSCs and BMP-2 accelerated the bone remodeling process [76].

Malek-Khatabi et al. evaluated the effects of the microfluidic-assisted synthesis of plasmid DNA (pDNA)-based chitosan nanocomplex platforms for bone tissue engineering. In particular, pDNA encoding for human BMP-2 was used. The nanocomplexes were immobilized on a nanofibrous PCL scaffold functionalized with metalloprotease-sensitive peptides. In a rat calvarial defect model, the implantation of MSCs into loaded PCL membranes demonstrated a significant increase in the regenerated bone volume, and this composite induced the formation of more dense bone-like structures [77].

These studies indicated that the addition of biomolecules improved bone formation, enhancing the efficacy of the scaffolds enriched with MSCs. The addition of BMPs, especially, was tested in different models. Indeed, BMPs play a major role in bone regeneration [78].

### 4.4. In Vivo Studies Using Scaffolds Enriched with Genetically Modified or Pre-Treated MSCs for Bone Regeneration

Another strategy to enhance bone formation may be to genetically modify or pretreat MSCs. An overview of the studies described in this section is available in Table 4. A bioactive glass nanoparticle (BGN) system can be used for the gene delivery of BMP-2 pDNA into MSCs that, in turn, target the bone. The MSCs transfected with BGN containing BMP-2-pDNA were delivered to the calvarium defects through a Col gel. The results indicated an improvement in bone regeneration. Specifically, improvements in bone volume, bone surface area, and surface density compared with MSCs treated with gene-free BGN were observed. However, MSCs treated with gene-free BGN also showed some bone formation, indicating that the BGN alone can, at least in part, stimulate the osteogenic development of MSCs [79].

A new approach to increase bone formation was tested by genetically modifying BMSCs for OPG delivery using an HA scaffold in critical-sized mandibular bone defects in ovariectomy-induced osteoporotic rats. The genetically modified BMSCs group showed the greatest level of mineralized new bone both at four and eight weeks, followed by the group with unmodified cells. On the contrary, HA alone only showed small amounts of new bone. Interestingly, the HA-OPG-BMSC constructs reduced osteoclastogenesis [80]. The combined use of DMP1-transduced BMSCs and Bio-Oss was shown to promote new bone formation and osseointegration in maxillary sinus floor augmentation implants in dogs [81]. Calcium phosphate cements (CPCs) are promising for dental repairs. Wang et al. developed an injectable cell delivery system based on the encapsulation of induced pluripotent stem cell-derived MSCs (iPSMSCs) in alginate microbeads, which were, in turn, dispersed in CPC. Specifically, iPSMSCs were pre-osteoinduced for two weeks or transduced with BMP-2. In vivo, the formation of the new bone area fraction was better when iPSMSCs transduced with BMP-2 were used, followed by pre-osteoinduced iPSMSCs. Cell-CPC constructs accelerated scaffold resorption [82].

It was found that a type I Col sponge containing BMSCs with *N*-acetyl-L cysteine (NAC) treatment implanted in critical-sized rat femur defects reduced the number of apoptotic cells while increased surviving cells at the transplantation site. Moreover, new bone formation was enhanced. These data suggested that pre-treatment of BMSCs with NAC before transplantation enhanced bone regeneration, increasing resistance to oxidative stress-induced apoptosis at the transplantation site [83].

In order to study the role of miRNA-21 in osteogenesis, miRNA-21-modified BMSC/β-TCP composite scaffolds were implanted into canine alveolar bone critical-sized defects. A greater volume of new bone formation was found in the miRNA-21 group compared to the control group, showing that miRNA-21 had a main role in inducing bone regeneration [84].

A study combined inducible transgene expression and near infrared (NIR)-responsive hydrogels technologies with the aim to develop a new strategy for bone regeneration. MSCs were genetically engineered in order to obtain the heat-activated and dimerizer-dependent transgene expression of BMP-2. These MSCs were seeded on hydrogels made of fibrin and plasmonic gold nanoparticles that transduced the incident energy of an NIR laser into heat. When a dimerizer was present, photoinduced mild hyperthermia led to the release of bioactive BMP-2 from the NIR-responsive cell constructs. In animals that were treated with a dimerizer, the NIR irradiation of implants induced BMP-2 production in the bone lesion, resulting in the formation of new mineralized tissue. Interestingly, 10 weeks after implantation, no animals showed traces of the hydrogels [85]. The regeneration of cementum and periodontal ligaments through the use of PDLSC sheet engineering technology with the pretreatment of recombinant human BMP-2 (rhBMP-2) was evaluated. Specifically, PDLSCs were pretreated with rhBMP-2, grafted onto micro/macro-porous biphasic calcium phosphate (MBCP) blocks, and finally implanted in mice. Interestingly, PDLSCs were viable for four weeks. rhBMP-2-pretreated hPDLSC sheets showed greater mineralized tissue formation and Col ligament deposition compared to not pretreated cells. Moreover, pretreated hPDLSC sheets promoted cementum-like mineralized structure formation [86].

Fan et al. induced the overexpression of Tribbles homolog 3 (Trb3), which plays a role in cell differentiation, in MSCs. The in vivo transplantation of MSCs overexpressing Trb3 seeded on an apatite/PLGA scaffold induced bone regeneration in a calvaria defect model [87].

These studies evidenced that genetically modified MSCs may be more useful than native MSCs in association with scaffolds for bone regeneration. Indeed, both genetic modification and pretreatment can improve MSC features or induce the expression of favorable growth factors.

### 4.5. In Vivo Studies Using Scaffolds Enriched with MSCs from Different Sources for Bone Regeneration

Some studies also evaluated the different abilities of MSCs obtained from various sources in inducing bone regeneration. An overview of the studies described in this section is available in Table 5. Wang et al. investigated the use of platelet-rich fibrin (PRF) combined with PDLSC and JBMMSC sheets for periodontal tissue engineering. PDLSC sheet/PRF/JBMMSC sheet composites were positioned in a simulated periodontal space made of a human-treated dentin matrix and HA/TCP. Eight weeks after implantation, the PDLSC sheets developed into periodontal ligament-like tissues, while the JBMSC sheets tended to predominantly produce bone-like tissues. Blood vessel formation was observed in the newly formed tissues. Instead, no cementum-like structures were found. These findings suggested that PDLSC sheets tended to form fiber tissues, but JBMMSC sheets may be more prone to form new bone. Thus, their combination may induce new periodontal ligament and bone tissues for periodontal tissue regenerative approaches [88]. Freitas et al. evaluated the bone repair of rat calvarial defects using a poly(vinylidene-trifluoroethylene)/barium titanate (PVDF-TrFE/BT) membrane alone or one injected with BMSCs or ADMSCs differentiated toward osteoblastic cells. These derived osteoblastic cells were detected in bone defects after cell injection for 25 days. Osteoblastic cells from BMSCs with the PVDF-TrFE/BT membrane increased bone formation, bone volume, bone volume percentage, bone surface, and trabecular number, while those derived from ADMSCs were not able to enhance bone repair [89]. Another study evaluated the angiogenic and osteogenic potential of a CPC scaffold seeded with human umbilical vein endothelial cells (hUVECs) and MSCs from different origins. In particular, the different MSC types were: human umbilical cord MSCs (hUCMSCs), human BMSCs, MSCs from induced pluripotent stem cells (hiPSC-MSCs), and embryonic stem cells (hESC-MSCs). The new bone and blood vessel density of cocultured groups were greater than those of the CPC without cells and CPC with BMSCs. However, the coculture of hUVEC with hUCMSCs, hiPSC-MSCs, and hESC-MSCs showed new bone and vessel density similar to the coculture of hUVEC with BMSCs. The results suggested that hUCMSCs, hiPSC-MSCs, and hESC-MSCs may represent alternative cell sources to BMSCs [90]. Another scaffold made of polyvinyl alcohol (PVA)-PCL-HA-based bioceramic (HAB) seems to be promising in the dentistry field. PVA-PCL-HAB scaffold implantation did not cause inflammation in mice. Through histologic examination, it was possible to find areas of bone formation in implants enriched with BMSCs or DPSCs. Interestingly, PVA-PCL-HAB implants were vascularized [91].

In rats with a critical-sized defect, more new bone formation was found in the group implanted with PCL enriched with periosteum-derived MSCs (PMSCs) compared to a control, while no healing was observed when the scaffold was seeded with BMSCs. Interestingly, the PMSCs showed longer survival times than BMSCs [92]. Human DPSCs and BMSCs were evaluated together with anorganic bone mineral (ABM) coated with a biomimetic Col peptide (ABM-P-15) for their capacity to improve bone formation. DPSCs showed a better osteogenic capacity. ABM-P-15 promoted DPSCs’ osteogenic differentiation and bone matrix formation. DPSCs seeded on ABM-P-15 scaffolds showed the better formation of an organized collagenous matrix compared to ABM alone in vivo [93].

Additionally, the regenerative capacity of GMSCs and BMSCs loaded onto a NanoBone scaffold, formed by HA nano-crystalline particles embedded in an amorphous silica gel matrix, was evaluated and compared with an unseeded scaffold. The transplantation of GMSCs and BMSCs loaded onto the NanoBone showed better bone regeneration compared to a scaffold without cells. Interestingly, no difference was found in the new bone formed by the scaffolds loaded with GMSCs or BMSCs, indicating that GMSCs represent a good alternative to BMSCs [94].

These studies indicated that different MSC sources may be useful for bone regeneration. Specifically, these studies suggested that different MSC sources other than BMSCs, which require an invasive procedure for collection, can be used.

## 5. Future Perspective: Cell-Free Approach

In recent years, new research in tissue engineering had proposed the use of cell-free therapies in order to avoid the ethical concerns in the use of whole cells. Moreover, MSCs have shown a limited survival when transplanted in vivo [95,96,97]. As widely reported, MSCs synthesize and release many growth factors, proteins, and free nucleic acids into the CM [16,98]. MSCs’ secretome, both CM and EVs, can be effective in repairing bone defects, as well as in other regenerative fields [17,18,99]. Moreover, it was reported that PDLSC-derived CM contains various cytokines, including interleukins and TGF-β [100,101]. EVs and exosomes (EXOs) in particular may also play central roles in cell-free therapies. EVs are vesicles released by MSCs that contain proteins, lipids, mRNA, microRNA, and cytokines. These vesicles release their contents into target cells, modulating their activity and potentially inducing restorative processes [102]. In addition, EVs’ immunomodulatory effects makes them suitable for autologous and allogenic therapies [103]. Finally, EVs are easy to collect. In fact, they can be isolated from MSCs with different origins. OMSCs, such as GMSCs [104] and PDLSCs [105], produce EVs that have been shown to be useful in some models for regenerative therapies. Recent studies confirmed that MSC-derived EVs may be promising for bone regeneration [106,107]. MSC-derived EVs enhance osteoblastic differentiation, improve osteochondral regeneration, and allow for bone defect-healing [108]. Here, we reviewed the studies that evaluated bone regeneration in in vivo models using biomaterials enriched with the MSC secretome. With this aim, we performed a PubMed search using the keywords mesenchymal stem cell, conditioned medium, exosome, extracellular vesicle, and oral bone regeneration. Specifically, some molecules, such as MCP-1, IGF-1, VEGF, and TGF-β, may play central roles in bone regeneration. Indeed, CM depleted of these growth factors did not show beneficial effects in bone repair, while a mixture of these factors showed similar effects to CM [109,110]. An overview of the studies described in this section is available in Table 6.

An atelocollagen scaffold with CM induced new bone formation in a rat calvarial bone defect model. The CM was shown to contain IGF-1, VEGF, and TGF-β1. Interestingly, when CM with anti-VEGF antibodies was used, bone regeneration was less efficacious. Indeed, both blood vessels and the migration of endogenous stem cells were enhanced by normal CM. The results indicated that VEGF is a main factor for bone regeneration with angiogenesis [111].

Hwang et al. evaluated the effects on the bone regeneration of CM derived from MSCs cultivated on a type I Col sponge with or without electrical stimulation. Both the constructs enhanced bone volume compared to the group that was implanted with MSCs. Moreover, in a model of bone loss induced by inflammatory cytokines, the group treated with a Col sponge with electrical stimulation showed better bone healing to levels comparable with MSCs [112].

Additionally, SHED-derived CM was shown to enhance bone regeneration. In a study where stem cell- and CM-containing atelocollagen were implanted, bone regeneration was evident in the defects treated with both stem cells and CM. However, bone regeneration was more prominent with CM, and mature bone formation and angiogenesis were found only in the CM group. Interestingly, the CM was shown to contain factors correlated to angiogenesis such as VEGF, and osteogenesis, including OPG, OPN, BMP-2, and BMP-4 [113].

Ogisu et al. evaluated the osteogenic activity of CM obtained from BMSCs cultured under cyclic stretch stimulation. CM in a collagen sponge was administered to a mouse calvarial defect model, and the results showed that bone regeneration and angiogenesis were enhanced by CM obtained from the cyclic stretch culture group. Indeed, CM obtained from cells cultured in the cyclic stretch stimulation contained more BMP-2, BMP-4, and VEGF-A [114].

CM derived from GMSCs and PDLSCs loaded onto a Col membrane were able to regenerate periodontal tissue. Newly formed bone in the periodontal defects was observed with an increased number of Runx2-positive cells. PDLSC-CM and GMSC-CM were also able to reduce inflammation, decreasing the number of cells positive for TNF-α and IL-1β while increasing those positive for IL-10, especially in the GMSC-CM group [115].

Katagiri et al. evaluated the effects of a cytokine cocktail that mimics the CM on the bone regeneration. The cocktail consisted of recombinant human insulin-like growth factor-1, VEGF-A, and TGF-β1 in concentrations similar to those found in the CM. The cocktail was tested for its bone regeneration capacity with an atelocollagen sponge in a rat calvarial bone defect model. In parallel, also CM and CM depleted from these cytokines were evaluated. Interestingly, both the cytokine cocktail and CM enhanced bone regeneration thanks to the recruitment of endogenous stem cells and endothelial cells. On the contrary, cytokine-depleted CM was not efficacious [110]. The same mixture was efficacious for periodontal tissue repair in class II furcation defects in dogs [130].

A cell-free bone tissue engineering system was created through the combination of ADMSC-derived EXOs and a PDA-coating PLGA scaffold. The construct was able to increase bone regeneration through its osteoinductive effects and the capacity to promote MSC migration and homing into the new bone [116].

In a rat model of periodontal intrabony defects, an EXO-loaded Col sponge repaired bone defects, increasing new bone formation. Interestingly, the regeneration of periodontal tissues and periodontal ligament (PDL) improved. Cellular infiltration and proliferation also increased in rats treated with the EXO Col sponge. The results suggested that MSC EXOs enhanced regeneration through an increase in cell mobilization and proliferation [117].

Additionally, EXOs enriched with miR-375, generated from ADMSCs overexpressing miR-375, were tested in vivo. A hydrogel consisting of thiol-modified hyaluronan, HA, and thiol-modified heparin was combined with EXOs enriched with miR-375. The construct enhanced bone regeneration more efficiently than normal EXOs [118].

Moreover, it was reported that EXOs can induce angiogenesis together with bone regeneration. Indeed, EXOs implanted in a rat model of calvaria bone defect with an atelocollagen sponge increased bone formation at two and four weeks after implantation. Additionally, CM induced positive effects on bone regeneration. In parallel, the accumulation of osteoblast-like cells and vascular endothelial cells was found. Interestingly, when EXOs with an angiogenesis inhibitor was used, bone regeneration failed [119].

EXOs derived from DPSCs encapsulated in tri-block PLGA–PEG–PLGA microspheres incorporated into a nanofibrous PLLA scaffold stimulated bone tissue regeneration in vivo without the need for exogenous stem cell transplantation. Scaffolds containing EXOs from mineralizing DPSCs showed the best results: a Col-rich matrix, new bone tissue, and integration with the host tissue [120].

The BMSC-derived small EVs loaded with hydrogel, composed of gelatin blended with Laponite, were administered into an experimental periodontitis rat model. The results showed that alveolar bone loss, inflammatory infiltration, and collagen destruction diminished in the small EV-hydrogel group. Their mechanism of action involved the OPG-RANKL-RANK pathway [121].

With the aim to increase osteoinductive abilities, MSCs were genetically modified to constitutively express BMP-2, with the hypothesis that the derived EVs showed enhanced osteoinductive properties. The derived EVs, loaded onto a collagen tape, showed an increased bone regenerative potential compared to native EVs in a rat calvarial defect model. However, it was shown that these EVs did not contain BMP-2, suggesting that they potentiate the BMP-2 signaling cascade, probably altering miRNA composition [122].

EXOs produced by BMSC in the osteoinductive condition was loaded onto a mesoporous bioactive glass (MBG) scaffold, where it enhanced new bone formation in a calvaria defect model [123].

Functionally engineered EVs were isolated from the BMP-2-expressing MSCs, loaded onto an alginate hydrogel linked with the RGD domain of fibronectin and implanted in a calvarial defect model. After eight weeks, this construct induced an enhanced bone regeneration when compared to the scaffold alone or without the RGD domain [124].

However, some studies also evaluated secretome in the presence of MSCs, demonstrating an enhancing role. Our group evaluated the osteogenic ability of a PLA scaffold enriched with GMSCs, EVs, or polyethyleneimine (PEI)-engineered EVs (PEI-EVs). The scaffold containing PEI-EVs, with or without cells, implanted in rats subjected to cortical calvaria bone tissue damage was able to improve bone healing, ultimately showing better osteogenic properties [125]. In addition, a PLA scaffold enriched with GMSCs and CM also showed a good osteogenic capacity, repairing the calvaria defect in a rat model [126]. The bone regeneration capacity of a Col membrane enriched with human PDLSCs and CM, EVs, or PEI-EVs was evaluated in rats subjected to calvarial defects. Rats implanted with Col, PDLSCs, and PEI-EVs showed increased bone regeneration in association with vascularization. In parallel, increases of VEGF and VEGF receptor 2 (VEGFR2) were observed [127].

Additionally, the evolution membrane enriched with PDLSCs and CM showed the good osteogenic ability to repair calvarial defects [128]. A construct formed by a PLA scaffold enriched with GMSCs and their EVs activated bone regeneration and vascularization in a rat calvarial defect model. The upregulation of miR-2861 and -210, other than RUNX2, VEGFA, OPN, and COL1A1, may explain the results [129].

Given the emergence of biomaterials enriched with CM or MSC-derived EVs as an auspicious alternative to cellular therapies, future studies to uncover the beneficial roles of CM and EVs on all regenerative processes are warranted to identify suitable tissue regenerative alternatives in patients.

Interestingly, the first study on patients has already been carried out. In particular, alveolar bone regeneration was evaluated in patients treated with β-TCP or an atelocollagen sponge soaked with CM, showing bone formation. Of note, no systemic or local complications were reported [131]. Additionally, promising results were obtained with β-TCP soaked with BMSC-derived CM for maxillary sinus floor elevation [132].

## 6. Conclusions

Tissue engineering has shown promising results for bone regeneration. This review shows the role of different biomaterials together with MSCs of different origins in tissue engineering processes. Indeed, unseeded scaffolds have shown limited regenerative potential. Moreover, the bone regenerative potential of scaffolds enriched with MSCs can be influenced and improved by the addition of biomolecules, such as BMPs, or the modulation of biomaterial features, such as pore dimension. It is important to notice that most of the studies used composite scaffolds or biomaterials with surface modifications, together with MSCs. MSCs obtained from different sources in association with scaffolds have been shown to enhance tissue regeneration and may be helpful when bone repair is required. BMSCs are the most used, but other MSCs sources also seem useful, including OMSCs that present the advantage of an easier isolation. In addition, constructs obtained using pre-treated or genetically modified MSCs can also help bone regeneration, thus improving bone formation. Cell-free therapies, through the secretome produced by MSCs, have also shown beneficial effects in bone regeneration, and so they represent a promising approach in this field. Moreover, further studies on these new therapies will be beneficial to develop promising tissue repair alternatives for patients.

## Figures and Tables

**Table 1:** Overview of the studies evaluating bone regeneration combining MSCs and scaffold in in vivo models.

Biomaterial	MSCs	Species	Model	Results	Ref.
C/ABB scaffold	JBMMSCs	Beagle dogs	Infrabony defects	New bone and cementum formation	[49]
PDA-laced HCCS	Osteogenic aggregated BMSCs	Sprague Dawley rats	Critical-sized calvarial defects	New bone formation was visible in scaffold with MSCs, while the scaffold without MSCs showed limited osteoconductivity	[50]
Bio-Oss^®^	BMSCs	Beagle dogs	Maxillary sinus floor augmentation	Newly formed bone in groups treated with Bio-Oss^®^ and BMSCs may be more mature	[51]
Laponite^®^ crosslinked with NIPAM and DMAc loaded with HA nanoparticles	BMSCs	Wistar rats	Femur defect model	In young male rats, bone defect repair seemed to be less effective when l-pNIPAM-co-DMAc seeded with MSCs was injected. In aged rats, an increase in runx2 was found in rats treated with l-pNIPAM-co-DMAC with HAna and MSC, suggesting an improved osteogenicity	[52]
PCL, Col, and nano-HA	BMSCs	Mice	Femur defect model	Combined with a bone allograft restored donor-site periosteal bone formation, reversing the poor biomechanics of bone allograft healing	[53]
Matrigel	Tonsil-derived MSCs	Sprague Dawley rats	Osteoradionecrosis	Bone regeneration and mineral density were better in the group transplanted immediately after trauma	[54]
PCL	Gingival cells, BMSCs, and PDLC	Sheep	Periodontal defect model	The PDLC group showed an enhanced bone formation	[55]
Fibrinogen and gelatin	Endothelial cells and MSCs	Sprague Dawley rats	Critical-sized calvarial defects	When the cells in the scaffolds were separated by a distance of <200 μm, an elevated number of blood vessels was shown and major bone regeneration	[56]
HA	SHED	Wistar rats	Alveolar bone defect model	Improved alveolar bone defect regeneration	[57]
HA matrix with PLGA	DPSCs	New Zealand rabbits	Bilateral mandibular critical-sized defects	Induced new bone formation and angiogenesis. The scaffold without DPSCs was less efficacious	[58]
TCP–PLGA scaffolds	Osteogenic differentiated ADMSCs	Miniature pigs	Mandibular defect model	Increased bone volume and osteocalcin deposition; the scaffold without cells was less efficacious	[59]
Alginate-based hydrogel modified with dopamine and methacrylate residues and with HA microparticles	GMSCs	Beige nude XID III (nu/nu) mice; Sprague Dawley rats	Subcutaneous model; Peri-implantitis model	Induced a complete bone regeneration	[60]
Self-crosslinking thiolated hyaluronic acid/type I Col I blend hydrogel and biphasic calcium phosphate ceramics	BMSCs	New Zealand rabbits	Osteochondral defect model	The BMSCs/chondrocyte scaffold promoted bone regeneration	[61]

ADMSCs: adipose-derived MSCs; BMSCs: bone marrow-derived MSCs; C/ABB: chitosan/anorganic bovine bone; Col: collagen; DMAc: *N*,*N*′–dimethylacrylamide; DPSCs: dental pulp stem cells; GMSCs: gingival MSCs; HA: hydroxyapatite; HCCS: HA Col calcium silicate; HAna: HA nanoparticles; JBMMSCs: jaw bone marrow-derived MSCs; MSCs: mesenchymal stem cells; NIPAM: *N*-isopropylacrylamide; PCL: polycaprolactone; PDA: polydopamine; PDLC: periodontal ligament cells; PLGA: polylactic polyglycolic acid; PVA: polyvinyl alcohol; SHED: stem cells from human exfoliated deciduous teeth; TCP: tri-calcium phosphates.

**Table 2:** Overview of the studies comparing different scaffolds enriched with MSCs for bone regeneration in in vivo models.

Biomaterial	MSCs	Species	Model	Results	Ref.
Polyamide, PLGA, or decellularized amniotic membrane	ADMSCs	Rabbits	Calvarial defects	Polyamide scaffolds showed the best results	[62]
PLDL, PDL, and HA/TCP	DPSCs	Immunocompromised mice	Subcutaneous implantation	PLDL, PDL, and HA-TCP enriched with DPSC seemed to be promising scaffolds for odontogenic regeneration	[63]
Col membrane with and without TCP material	BMSCs	Sprague Dawley rats	Critical-sized calvarial defects	The hardness of the new bone was similar to the native bone in groups with the Col membrane with and without cells. The bone in the group treated with Col membrane, TCP, and BMSCs showed a greater elasticity	[64]
20% HA/80% TCP, 60% HA/40% TCP, or Bio-Oss	ABMSCs	Mice	Ectopic transplantation model	ABMSCs with both HA/TCP scaffolds increased bone regeneration; Bio-Oss did not induce new bone formation when loaded with ABMSCs	[65]
Alginate hydrogels containing BMP-2	SHED	C57BL/6 mice	Subcutaneous	Scaffold with smaller pores and greater elasticity was found to potentially induce greater bone regeneration	[66]
Nanofibrous PLLA scaffolds with different pore sizes	BMSCs	Mice	Subcutaneous implantation	Bone volume increased with the increase of pore size	[67]
PDA-laced HCCS scaffolds with different pore dimensions	MSCs	Sprague Dawley rats	Critical-sized calvarial defects	New bone formation was observed after the implantation of the scaffold with pores of the size of 500 μm; instead, the scaffolds with 250 μm pores induced only a minimal bone formation	[68]
βTCP with or without nano-diamond particles	BMSCs	Merino sheep	Lateral augmentation of the mandible	βTCP with nano-diamond particles induced more bone formation; MSC addition resulted in little difference	[69]
HA-based bioceramic scaffold 10% of strontium	BMSCs	NOD.CB17-Prkdc^SCID^/Jmice; sheep	Ectopic bone formation; femoral critical-sized defects	The scaffolds containing 10% of strontium induced bone formation and osteogenic differentiation of MSC	[70]

ADMSCs: adipose-derived MSCs; BMP: bone morphogenetic protein; BMSCs: bone marrow-derived MSCs; DPSCs: dental pulp stem cells; HA: hydroxyapatite; HCCS: HA Col calcium silicate; MSCs: mesenchymal stem cells; PDA: polydopamine; PDL: copolymer of DL-lactide; PLDL: l-lactide and DL-lactide; PLGA: polylactic polyglycolic acid; PLLA, poly(l-lactic acid); SHED: stem cells from human exfoliated deciduous teeth; TCP: tri-calcium phosphates.

**Table 3:** Overview of the studies using scaffolds enriched with MSCs and biomolecules for bone regeneration in in vivo models.

Scaffold	MSCs	Enrichment	Species	Model	Results	Ref.
TCP scaffolds	BMSCs with endothelial progenitor cells	PLGA microspheres releasing VEGF	Mongrels	Mandibular defects	Bone formation was greatest in the VEGF/MSC scaffold, followed by the VEGF/MSC/EPC and MSC/EPC scaffolds	[71]
Alginate-chitosan beads	MSCs	BMP-2 or basement membrane proteins	Mice	Cranial defect	Induced bone repair	[72]
Nano-HA (nHA)/Gel/Gel microsphere	BMSCs	BMP-6	Sprague Dawley rats	Critical-sized calvarial defects	The new bone was larger with the BMP-6-loaded scaffolds	[73]
nHA/Col I/multi-walled carbon nanotube	BMSCs	BMP-9	Sprague Dawley rats	Critical-sized calvarial defects	The enrichment with BMP-9 increased bone formation	[74]
Heparin-conjugated Col hydrogel reinforced by 3D printed β-TCP-based bioceramic	DPSCs	BMP-2	Male Fischer 344 rats	Subcutaneous implantation	A greater new bone formation was found when heparin was present. BMP-2 increased the expression of genes involved in osteogenesis	[75]
PCL biomembranes	BMSCs	BMP-2	Nude mice	Maxillary bone lesion	BMSCs and BMP-2 accelerated the bone remodeling process	[76]
Nanofibrous PCL scaffold	BMSCs	pDNA encoding for human BMP-2	Sprague Dawley rats	Calvarium defect	Increased the regenerated bone volume, and this composite induced the formation of more dense bone-like structures	[77]

BMP: bone morphogenetic protein; BMSCs: bone marrow-derived MSCs; Col: collagen; DPSCs: dental pulp stem cells; Gel: gelatin; HA: hydroxyapatite; PCL: polycaprolactone; pDNA: plasmid DNA; TCP: tri-calcium phosphates.

**Table 4:** Overview of the studies using scaffolds enriched with pre-treated or genetically modified MSCs for bone regeneration in in vivo models.

Scaffold	MSCs	Species	Model	Results	Ref.
Col gel	BMSCs transfected with BMP-2 plasmid DNA	Sprague Dawley rats	Calvarium critical-sized defect	Improved in bone regeneration	[79]
HA scaffold	BMSCs for OPG delivery	Sprague Dawley rats	Critical-sized bone defects were created in the rat mandibles	The genetically modified BMSCs group showed the greatest level of mineralized new bone	[80]
Bio-Oss	DMP1-transduced BMSCs	Beagles	Maxillary sinus floor augmentation	Promoted of new bone formation	[81]
Calcium phosphate cements	Pre-osteoinduced or BMP-2 transduced iPSMSCs in alginate microbeads	Nude rats	Cranial bone defects	New bone area fraction was greater when iPSMSCs transduced with BMP-2 were used, followed by pre-osteoinduced iPSMSCs	[82]
Col sponge	BMSCs pre-treatment with *N*-acetyl-L cysteine	Sprague Dawley rats	Femur bone defect	Pre-treatment of BMSCs with NAC before transplantation enhanced bone regeneration	[83]
β-TCP	miRNA-21-modified BMSCs	Labrador dogs	mandibular defect model	A greater volume of new bone formation was found in the miRNA-21 group compared to the control group	[84]
Hydrogels made of fibrin and plasmonic gold nanoparticles	BMP-2-expressing MSCs	C3H/HeNRj mice	Critical-sized calvarial defects	Formed of new mineralized tissue.	[85]
Biphasic calcium phosphate (MBCP) blocks	PDLSC pretreatment of recombinant human BMP-2	BALB/c nude mice	Subcutaneous transplantation	rhBMP-2 pretreated hPDLSC sheets showed greater mineralized tissue formation and Col ligament deposition compared to not pretreated cells	[86]
Apatite/PLGA scaffold	Trb3 overexpressing MSCs	CD-1 nude mice; Sprague Dawley rats	Calvarial defect model; critical-sized mandible defects	Induced bone regeneration	[87]

BMP: bone morphogenetic protein; BMSCs: bone marrow-derived MSCs; Col: collagen; DMP1: dentin matrix protein-1; HA: hydroxyapatite; iPSMSCs: induced pluripotent stem cell-derived MSCs; OPG: osteoprotegerin; PDLSCs: periodontal ligament stem cells; PLGA: polylactic polyglycolic acid; TCP: tri-calcium phosphates; Trb3: Tribbles homolog 3.

**Table 5:** Overview of the studies that compared different MSC sources in combination with scaffold for bone regeneration in in vivo models.

Scaffold	MSCs	Species	Model	Results	Ref.
Dentin matrix and HA/TCP	platelet-rich fibrin PDLSCs and JBMSCs	Nude mice	Simulated periodontal space comprising human treated dentin matrix and HA/TCP frameworks	PDLSC sheets developed into periodontal ligament-like tissues, while the JBMSC sheets tended to predominantly produce bone-like tissues	[88]
PVDF-TrFE/BT membrane	BMSCs or ADMSCs differentiated toward osteoblastic cells	Wistar rats	Calvarial defect	Osteoblastic cells from BMSCs with the PVDF-TrFE/BT membrane increased bone formation, bone volume, bone volume percentage, bone surface, and trabecular number, while those derived from ADMSCs were not able to enhance bone repair	[89]
CPC scaffold	hUVECs and hUCMSCs, BMSCs, hiPSC-MSCs, or hESC-MSCs	Rats	Critical-sized cranial defect	The coculture of hUVEC with hUCMSCs, hiPSC-MSCs, and hESC-MSCs showed new bone and vessel density similar to the coculture of hUVEC with BMSCs.	[90]
PVA-PCL-HA-based bioceramic	BMSCs or DPSCs	NOD-SCID mice	Ectopic bone formation	New bone formation was found	[91]
PCL	PMSCs and BMSCs	Sprague Dawley rats	Femoral critical-sized bone defect	New bone formation was found in the group implanted with the PMSC-enriched scaffold, while no healing was observed when the scaffold was seeded with BMSCs	[92]
Anorganic bone mineral coated with a biomimetic Col peptide (ABM-P-15)	DPSCs and BMSCs	MF1 Nu/Nu mice	Intraperitoneal transplantation	DPSCs showed a better osteogenic capacity	[93]
NanoBone scaffold	GMSCs and BMSCs	New Zealand rabbits	Tibiae bone defects	The transplantation of GMSCs and BMSCs loaded onto the NanoBone showed better bone regeneration compared to the scaffold without cells. Interestingly, no difference was found in the new bone formed by the scaffolds loaded with GMSCs or BMSCs	[94]

ADMSCs: adipose-derived MSCs; BMSCs: bone marrow-derived MSCs; CPC: calcium phosphate cements; DPSCs: dental pulp stem cells; GMSCs: gingival-derived MSCs; HA: hydroxyapatite; PCL: polycaprolactone; PVA: polyvinyl alcohol; PVDF-TrFE/BT: poly(vinylidene-trifluoroethylene)/barium titanate; TCP: tri-calcium phosphates.

**Table 6:** Overview of the studies using the MSC secretome in combination with biomaterial for bone regeneration in in vivo models.

Secretome	MSC Source	Biomaterial	Species	Model	Results	Ref.
CM or cytokine cocktail that mimics the CM	BMSCs	Atelocollagen sponge	Wistar/ST rats	Calvarial bone defects	Bones regenerated thanks to the recruitment of endogenous stem cells and endothelial cells	[110]
CM	MSCs	Atelocollagen	Wistar/ST rats	Calvarial bone defect model	New bone formation	[111]
CM	BMSCs	Col sponge	Sprague Dawley rats; BALB/C mice	Calvaria defect model; inflammatory bone loss	Enhanced bone volume	[112]
CM	SHED	Atelocollagen sponge	Deficient mice (BALB/c-nu)	Calvarial bone defect model	Enhanced bone regeneration and angiogenesis	[113]
CM	BMSCs cultured under cyclic stretch stimulation	Col sponge	Mice	Calvarial defect model	Bone regeneration and angiogenesis were enhanced by CM obtained from the cyclic stretch culture group	[114]
CM	GMSC and PDLSC	Col membrane	Wistar rats	Periodontal defect model	Newly formed bone and reduced inflammation	[115]
EXO	Adipose-derived stem cells	Polydopamine-coating PLGA	BALB/C mice	Calvarial critical-sized defect	Promoted MSC migration and homing into the new bone	[116]
EXO	MSCs	Col/III sponges	Sprague Dawley rats	Periodontal defect model	Regenerated bone and periodontal tissues	[117]
EXO overexpressing miR-375	Adipose-derived stem cells overexpressing miR-375	Hydrogel consisting of thiol-modified hyaluronan, HA and thiol-modified heparin	Sprague Dawley rats	Calvarial defects	Enhanced bone regeneration	[118]
EXO	BMSCs	Atelocollagen sponges	Wistar rats	Calvarial critical-sized defect	Bones regenerated and angiogenesis occurred	[119]
EXO	DPSCs	Tri-block PLGA–PEG–PLGA micro-spheres incorporated into a nanofibrous PLLA scaffold	C57BL/6 mice	Calvarial Defect	Bone tissue regenerated	[120]
Small EVs	BMSCs	Gelatin blended with Laponite	Sprague Dawley rats	Periodontitis rat model	Alveolar bone loss, inflammatory infiltration, and collagen destruction diminished	[121]
EVs	BMSCs were genetically modified to constitutively express BMP-2	Collagen tape	Rats	Calvarial bone defect	Increased bone regenerative potential	[122]
EXO	BMSC in osteoinductive condition	Mesoporous bioactive glass	Rats	Critical-sized calvarial defect	New bones formed and regenerated	[123]
EVs	BMP 2 expressing MSCs	Alginate hydrogel linked with the RGD domain of fibronectin	Rats	Calvarial bone defect model	Enhanced bone regeneration	[124]
EVs, or PEI-engineered EVs	GMSCs	PLA	Wistar rats	Calvarial defect	The scaffold containing PEI-EVs, with or without cells, were able to improve bone healing	[125]
CM	GMSCs	PLA scaffold	Wistar rats	Calvarial defect	Good osteogenic capacity was observed	[126]
CM, EVs, or EVs engineered with PEI	PDLSCs	Col membrane	Wistar rats	Calvarial defect	Increased bone regeneration in association with vascularization	[127]
CM	PDLSCs	Evolution membrane	Wistar rats	Calvarial defect	Good osteogenic ability was observed	[128]
EVs	GMSCs	PLA	Wistar rats	Calvarial defect	Bone regeneration and vascularization were observed	[129]

BMP: bone morphogenetic protein; BMSCs: bone marrow-derived MSCs; CM: conditioned medium; Col: collagen; DPSCs: dental pulp stem cells; EVs: extracellular vesicles; EXO: exosomes; GMSCs: gingival-derived MSCs; HA: hydroxyapatite; HCCS: HA Col calcium silicate; MSCs: mesenchymal stem cells; PDLSCs: periodontal ligament stem cells; PEI: polyethylenimine; PLA: polylactic acid; PLGA: polylactic polyglycolic acid; SHED: stem cells from human exfoliated deciduous teeth; TCP: tri-calcium phosphates.

## Data Availability

Not applicable.

## Abbreviations

MSCs	Mesenchymal stem cells
ECM	Extracellular matrix
BMSCs	Bone marrow-derived MSCs
OMSCs	Oral mesenchymal stem cells
DPSCs	Dental pulp stem cells
SCAPs	Stem cells from the apical papilla
PDLSCs	Periodontal ligament stem cells
GMSCs	Gingival-derived MSCs
DFSCs	Dental follicle stem cells
TGSCs	Tooth germ stem cells
ABMSCs	Alveolar bone-derived MSCs
CM	Conditioned medium
EVs	Extracellular vesicles
VEGF	Vascular endothelial growth factor
TGF-β	Transforming growth factor β
BMPs	Bone morphogenetic proteins
ESB	European Society for Biomaterials
HA	Hydroxyapatite
β-TCP	Β-tricalcium-phosphate
Col	Collagen
PLA	Polylactic acid
PCL	Polycaprolactone
PLLA	Poly(l-lactic acid)
PDLA	Poly(d,l-lactic acid)
PLGA	Polylactic-co-glycolic acid
PGA	Polyglycolic acid
C/ABB	Chitosan/anorganic bovine bone
JBMMSCs	Jaw bone marrow-derived MSCs
PDA	Polydopamine
HCCS	HA Col calcium silicate
CGFs	Concentrated growth factors
NIPAM	*N*-isopropylacrylamide
DMAc N	*N*′–dimethylacrylamide
Hana	HA nanoparticles
TEP	Tissue-engineered periosteum
PDLCs	Periodontal ligament cells
SHED	Stem cells from human exfoliated deciduous teeth
OPG	Osteoprotegerin
ADMSCs	Adipose-derived MSCs
PLDL	l-lactide and dl-lactide
PDL	dl-lactide
DSPP	Dentin sialo-phosphoprotein
DMP1	Dentin matrix protein-1
MMP20	Enamelysin/matrix metalloproteinase 20
PHEX	Phosphate-regulating gene with homologies to endopeptidases on X Chromosome
SrHAB	Strontium-substituted HA-based bioceramic scaffold
EPCs	Endothelial progenitor cells
pDNA	Plasmid DNA
BGN	Bioactive glass nanoparticle
CPCs	Calcium phosphate cements
iPSMSCs	Induced pluripotent stem cell-derived MSCs
NAC	*N*-acetyl-l cysteine
Trb3	Tribbles homolog 3
PRF	Platelet-rich fibrin
PVDF-TrFE/BT	Poly(vinylidene-trifluoroethylene)/barium titanate
hUVECs	Human umbilical vein endothelial cells
hUCMSCs	Human umbilical cord MSCs
hiPSC-MSCs	MSCs from induced pluripotent stem cells
hESC-MSCs	Embryonic stem cells
PVA	Polyvinyl alcohol
HAB	HA-based bioceramic
PMSCs	Periosteum-derived MSCs
ABM	Anorganic bone mineral
EXO	Exosomes
MBG	Mesoporous bioactive glass
PEI	Polyethyleneimine
PEI-EVs	Polyethyleneimine-engineered EVs
VEGFR2	VEGF receptor 2
